# Cuckoo search algorithm based on cloud model and its application

**DOI:** 10.1038/s41598-023-37326-3

**Published:** 2023-06-21

**Authors:** Yan Xiong, Ziming Zou, Jiatang Cheng

**Affiliations:** College of Mechanical and Control Engineering, Guilin University of Technology, Guilin, 541006 China

**Keywords:** Engineering, Mathematics and computing

## Abstract

Cuckoo search algorithm is an efficient random search method for numerical optimization. However, it is very sensitive to the setting of the step size factor. To address this issue, a new cuckoo search algorithm based on cloud model is developed to dynamically configure the step size factor. More specifically, the idea of giving consideration to both fuzziness and randomness of cloud model is innovatively introduced into cuckoo search algorithm, and the appropriate step size factor can be determined according to the membership degree and an exponential function, so as to realize the adaptive adjustment of the control parameter. After that, simulation experiments are conducted on 25 benchmark functions with different dimensions and two chaotic time series prediction problems to comprehensively evaluate the superiority of the proposed algorithm. Numerical results demonstrate that the developed method is more competitive than the other five CS and several non-CS algorithms.

## Introduction

As a population-based evolutionary algorithm, cuckoo search (CS)^1^ has been favored by many scholars since it was proposed in 2009 due to its few parameters and strong global search ability. So far, CS algorithm has been successfully applied to solving various optimization problems in scientific and engineering fields^2–6^.

The core advantage of CS algorithm is that the Levy flight strategy is employed to generate new candidate solutions. The highly random flight mechanism allows individuals to explore the entire solution space as much as possible, so the population exhibits good diversity, but it also leads to poor local exploitation ability and slow convergence speed of the algorithm. Furthermore, when dealing with complex optimization problems, CS algorithm, like other evolutionary computation methods^7,8^, also has the problem of low search efficiency. Therefore, many scholars have conducted extensive research to enhance the convergence performance of the algorithm. At present, the improvement strategies of CS algorithm mainly include two categories: parameter control and hybridization^9^.

As for the parameter control, the adaptive scheme is a commonly used strategy. Reda et al.^10^ developed a double exponential CS algorithm, in which the discovery probability was adjusted adaptively according to the concept of the double Mersenne numbers. After that, the proposed algorithm and other CS variants were compared and evaluated on the CEC2017 benchmark functions. Wei and Yu^11^ designed a CS with adaptive parameter control. In this variant, the Cauchy distribution and Lehmer mean were used to dynamically update the control parameters. Then, 48 benchmark functions and two fractional-order chaotic systems were employed to verify the convergence performance. Mareli and Twala^12^ proposed three CS algorithms based on dynamically increasing conversion parameters. Also, these algorithms were tested on 10 benchmark functions. Simulation results indicated that CS with exponentially increasing conversion parameters is superior to the other two methods. Bulatović et al.^13^ presented an improved CS algorithm for constrained optimization problems. For this version, the step size factor and discovery probability changed dynamically with the number of generations. Also, four constrained engineering optimization problems were considered to test the effectiveness of this algorithm. In terms of hybridization with other methods, some attempts have been made to enhance the performance of CS algorithm. Khadanga et al.^14^ proposed a CS combined with grey wolf optimization (GWO) algorithm for load frequency controller design. First, 10 benchmark functions were used to test the superiority of this hybrid algorithm. Then, the proposed method was applied to the controller design of power system. Kumar et al.^15^ developed a hybrid algorithm based on CS and quantum behaved particle swarm optimization (QPSO). In this version, CS was modified by adjusting the step size factor. After that, the total population was divided into two parts, half of which was updated by CS and the other half by QPSO. Shehab et al.^16^ designed an improved CS by combining with bat algorithm (BA). In this hybrid method, two populations were considered. The first population was evolved using CS, and the best solution was transferred to the second population optimized by BA. Based on the above analyses, there is no doubt that the convergence performance of these modified CS algorithms can be enhanced from different aspects, but the algorithm structure or time complexity usually changes to varying degrees. Therefore, exploring a new CS algorithm still has positive practical significance.

Motivated by these observations, a new CS algorithm based on cloud model (CCS) is developed to strengthen the search accuracy and efficiency. In the traditional CS algorithm, the step size factor is a very important control parameter, which depends on the problem to be solved. When dealing with different types of optimization problems, the step size factor should be able to change with the change of the optimization problem, exhibiting fuzziness and randomness. This uncertainty can be represented by a cloud model. The cloud model is a new method of uncertain information processing, which can comprehensively consider randomness and fuzziness^17^. Therefore, the cloud model is introduced into CS algorithm to configure the step size factor. First, the step size factor is regarded as the cloud droplet, and the corresponding membership degree can be calculated according to the mathematical characteristics defined in the cloud model. Next, an exponential function is designed to adaptively determine the step size factor by using the membership degree. Obviously, CCS has the same algorithm structure as the traditional CS. Finally, 25 benchmark functions and two chaotic systems are employed to evaluate the advantages of CCS algorithm.

The contributions of this paper are as follows.

1. The step size parameter of CS algorithm is regarded as the cloud droplet, and the parameter set is seen as the cloud. Three numerical characteristics of cloud model are defined by using the individual fitness information in the population.
2. According to the fuzziness and randomness of cloud model, the membership degree corresponding to each cloud droplet is calculated, and an exponential function is designed to realize the configuration from the membership degree to step size factor.
3. Extensive experiments are conducted on 25 benchmark functions and two chaotic systems to evaluate the advantages and competitiveness of CCS algorithm.

The remaining sections of this paper are structured as follows. CS algorithm is introduced in section "CS algorithm", and the proposed CCS algorithm is described in section "CS algorithm based on cloud model". The experiments and results are provided in section "Experiments and results", and the conclusions are drawn in section "Conclusions".

## CS algorithm

Cuckoo search (CS) algorithm^1^ is a global optimization method, which is based on the social behavior idea of cuckoo breeding offspring. CS algorithm mainly includes two important strategies: Levy flight and biased random walk. During each iteration, Levy flight is used to find better nests to lay eggs. For CS algorithm, each nest represents a feasible solution, and the population size of cuckoo represents the number of solutions.

In an ideal state, the nest position update formula based on Levy flight is as follows:

1 $$ x_{i}^{t + 1} = x_{i}^{t} + \alpha \oplus L\left( \lambda \right) $$

2 $$ \alpha = \alpha_{0} \otimes \left( {x_{i}^{t} - x_{best} } \right) $$

where $x_{i}^{t + 1}$ represents the new solution, $x_{i}^{t}$ and $x_{best}$ denote the current solution and the best solution respectively, and $t$ indicates the current iteration number. $\oplus$ is entry-wise multiplication, $\alpha$ is the stepwise parameter that controls the moving step size of cuckoo, and $\alpha_{0}$ denotes the step size factor, which is usually set to 0.01. $L\left( \lambda \right)$ stands for a random search path, which can be expressed as:

3 $$ L\left( \lambda \right) = \frac{\varphi \times m}{{\left| n \right|^{1/\beta } }} $$

where $m$ and $n$ are two random numbers subjected to the normal distribution, $\beta$ is set to 1.5. $\varphi$ is defined as:

4 $$ \varphi = \left( {\frac{{\Gamma \left( {1 + \beta } \right) \times sin\left( {\pi \beta /2} \right)}}{{\Gamma \left( {\left( {1 + \beta } \right)/2} \right) \times \beta \times 2^{{\left( {\beta - 1} \right)/2}} }}} \right)^{1/\beta } $$

Another strategy of CS algorithm is the biased random walk. After the nest position is updated by using Levy flight, some solutions are discarded according to the discovery probability $p_{a}$, and the same number of new solutions can be regenerated by using the biased random walk, which is described as follows:

5 $$ x_{i}^{t + 1} = x_{i}^{t} + rnd \times \left( {x_{g}^{t} - x_{k}^{t} } \right) $$

where $rnd$ is a random number ranging from 0 to 1, ${x}_{g}^{t}$ and ${x}_{k}^{t}$ are two randomly selected solutions.

## CS algorithm based on cloud model

As mentioned above, the search strategy of CS algorithm mainly consists of Levy flight and biased random walk, combined with the greedy selection scheme to find promising solutions. In the early stage of the search, a larger step size factor is used to expand the search space, thereby increasing the population diversity. In the late stage of the search, the step size factor should be set to a smaller value to enhance the local search capability of the algorithm. However, in addressing complex multimode problems, due to the lack of parameter adaptation adjustment mechanism, the parameter configuration scheme based on decreasing step size factors may lead to the algorithm being trapped into local optima. Therefore, a new CS algorithm based on cloud model (CCS) is proposed to adaptively adjust the step size factor, so as to strengthen the ability of the algorithm to address complex optimization problems.

**Figure 1:**
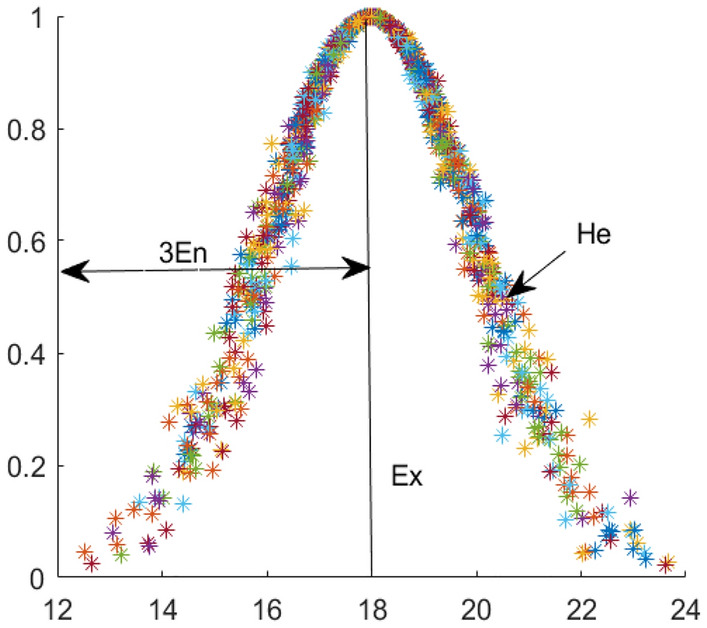
Schematic diagram of cloud model.

The cloud model is a cognitive model based on classical probability theory and fuzzy mathematics to realize the transformation of qualitative concepts and quantitative descriptions, reflecting the fuzziness and randomness of qualitative concepts^18^. The cloud model has three numerical characteristics, namely, expectation ${E}_{x}$, entropy ${E}_{n}$ and hyper entropy ${H}_{e}$. Expectation ${E}_{x}$ represents the information center value of cloud droplets, which is the central point of quantitative representation of qualitative concepts. Entropy ${E}_{n}$ represents the value range of qualitative concept, reflecting the dispersion degree and floating range of cloud droplets. The greater the entropy ${E}_{n}$, the more ambiguous the concept is. The hyper entropy ${H}_{e}$ is the uncertainty measure of entropy ${E}_{n}$, which reflects the degree of cloud dispersion. These three values together constitute the basic numerical characteristics of the cloud model, as shown in Fig. 1.

In this work, the cloud model is employed to produce the step size factor ${\alpha }_{0}$. First, the control parameter is regarded as a qualitative concept, the step size factor is considered as a cloud droplet, and the set of step size factors in the process of algorithm evolution is regarded as a cloud. Then, the expectation ${E}_{x}$, entropy ${E}_{n}$ and hyper entropy ${H}_{e}$ of the step size factor set can be obtained by using the forward cloud model. Finally, the membership degree of the step size factor is calculated according to the three numerical characteristics of the cloud model. In terms of the principle of the forward cloud model, the numerical characteristics can be expressed as:

6 $$ E_{x} = f\left( {best} \right) $$

7 $$ E_{n} = f\left( {avg} \right) - f\left( {best} \right) $$

8 $$ H_{e} = \frac{{E_{n} }}{5} $$

where $f\left( {best} \right)$ and $f\left( {avg} \right)$ correspond to the best fitness and average fitness of all individuals in the current population, respectively.

Based on these, the membership degree is defined as:

9 $$ \mu_{i} = \exp \left( {\frac{{ - \left( {f\left( {x_{i} } \right) - E_{x} } \right)^{2} }}{{2\sigma^{2} }}} \right) $$

where $\sigma$ represents the normal distribution random number with the mean $E_{n}$ and variance $H_{e}$, $f\left( {x_{i} } \right)$ is the fitness of the current individual.

To realize the reasonable setting of step size factor $\alpha_{0}$, an exponential function is employed to map the value of the membership degree $\mu_{i}$ to the ideal interval range, so as to adaptively configure $\alpha_{0}$. The exponential function is given as:

10 $$ \alpha_{0} = \frac{\gamma }{{1 + e^{{ - \mu_{i} }} }} $$

where $\gamma$ is a control coefficient to be preset.

Undoubtedly, the step size factor $\alpha_{0}$ is closely related to the individual fitness $f\left( {x_{i} } \right)$, which is conducive to addressing different types of optimization problems. The smaller the individual fitness, the greater the step size factor obtained, and vice versa. In other words, the step size factor of CCS algorithm can be adaptively configured according to the individual fitness, so it has good robustness. Therefore, this parameter adjustment strategy based on cloud model can alleviate the premature convergence or even stagnation of the algorithm.

## Experiments and results

### Benchmark functions

In the experiments, two sets of popular benchmark functions are chosen to evaluate the overall performance of the proposed CCS algorithm. The first set of functions contains 11 basic problems, as shown in Table 1. Their specific information can be found in literature^19^. The second set of functions includes 14 shifted and rotated problems, which are composed of the forehand 14 problems F1–F14 in the CEC 2005^20^. It's worth noting that these problems are very complex benchmark functions used to simulate real-world optimization problems.

**Table 1:** The first set of benchmark functions.

No.	Function name	Characteristic	Best value
f1	Sphere	Unimodal	0
f2	Schwefel 2.22	Unimodal	0
f3	Schwefel 1.2	Unimodal	0
f4	Schwefel 2.21	Unimodal	0
f5	Rosenbrock	Unimodal	0
f6	Schwefel 2.26	Multimodal	0
f7	Rastrigin	Multimodal	0
f8	Ackley	Multimodal	0
f9	Griewank	Multimodal	0
f10	Penalized 1	Multimodal	0
f11	Penalized 2	Multimodal	0

### Influence of the control coefficient

In the proposed CCS algorithm, there are two control parameters to be determined, namely the control coefficient $\gamma$ and discovery probability ${p}_{a}$. The discovery probability is set to the recommended value of 0.25. To evaluate the sensitivity of CCS performance to the control coefficient, additional experiments are conducted on the first set of benchmark functions with 30 dimensions. The population size of CCS algorithm is set to 50, the control coefficient $\gamma$ is set from 0.1 to 1, and the step size is set as 0.1. For each control coefficient, CCS is run 30 times on each test function. The maximum number of function evaluations is 300,000 to stop each run. The mean values of the final function errors are shown in Table 2, and the best result is highlighted in bold.

**Table 2:** Mean values obtained by CCS using different control coefficients.

No.	0.1	0.2	0.3	0.4	0.5	0.6	0.7	0.8	0.9	1.0
f1	6.4E−30	1.4E−38	**5.4E−41**	5.9E−40	4.9E−37	8.9E−34	4.3E−30	5.6E−27	1.8E−24	5.8E−22
f2	1.1E−15	7.7E−22	4.4E−24	**3.8E−24**	3.9E−23	1.2E−21	6.9E−20	5.6E−18	3.7E−16	1.7E−14
f3	**1.5E−11**	3.1E−11	3.5E−10	1.6E−09	7.7E−09	4.2E−08	1.8E−07	2.6E−07	1.0E−06	3.8E−06
f4	9.8E−03	4.9E−04	**1.9E−04**	**1.9E−04**	2.7E−04	5.3E−04	8.5E−04	1.5E−03	2.4E−03	3.5E−03
f5	6.1E+00	2.2E+00	**1.8E+00**	2.4E+00	3.7E+00	6.1E+00	7.7E+00	9.4E+00	1.1E+01	1.2E+01
f6	2.6E+03	2.4E+03	1.7E+03	1.4E+03	1.2E+03	9.4E+02	8.9E+02	7.5E+02	**7.1E+02**	8.1E+02
f7	3.7E+01	3.4E+01	3.0E+01	2.8E+01	2.9E+01	2.6E+01	**2.5E+01**	3.1E+01	**2.5E+01**	2.8E+01
f8	3.1E−14	1.1E−14	8.8E−15	8.1E−15	**8.0E−15**	8.1E−15	8.3E−15	1.9E−14	2.3E−13	3.6E−12
f9	**0.0E+00**	**0.0E+00**	2.5E−04	2.7E−16	9.6E−12	9.9E−04	9.0E−04	2.5E−04	4.9E−04	9.8E−04
f10	4.2E−24	**1.6E−32**	**1.6E−32**	**1.6E−32**	**1.6E−32**	7.8E−32	2.7E−28	1.6E−24	3.5E−03	1.4E−20
f11	6.8E−28	**1.3E−32**	**1.3E−32**	**1.3E−32**	**1.3E−32**	1.1E−31	3.1E−28	2.1E−24	9.2E−23	7.8E−20

Significant values are in bold.

From Table 2, it can be seen that the control coefficient $\gamma$ has a significant impact on the convergence accuracy of CCS algorithm. For example, CCS produces the better performance with a larger control coefficient on f6 and f7. On the contrary, CCS performs better on f3 and f9 in the case of a smaller control coefficient. Besides, for other benchmark functions, as the control coefficient $\gamma$ increases, the search accuracy of CCS algorithm increases first and then decreases. Obviously, the appropriate setting of control coefficient can have a satisfactory effect on the search capability. According to the experimental results, it is observed that the trade-off interval of the control coefficient $\gamma$ is from 0.2 to 0.5.

### Comparison with CS algorithms

**Figure 2:**
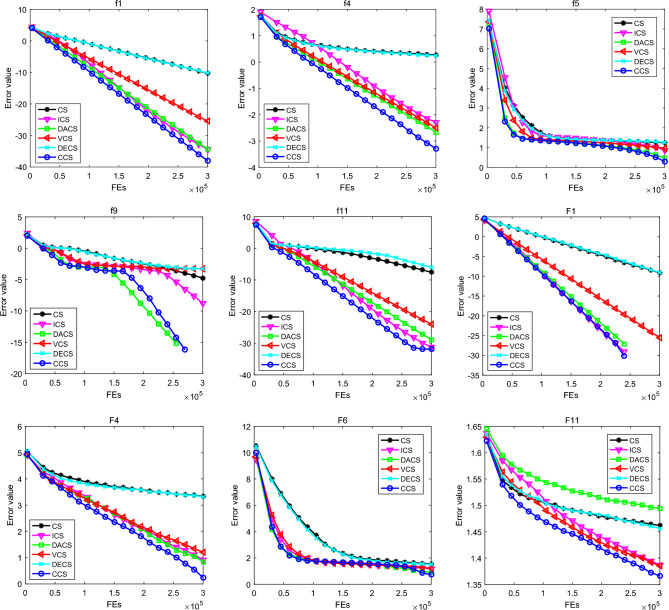
Convergent graphs of CCS and its competitors for the 30 dimensional benchmark functions.

To assess the advantages of CCS, five CS algorithms are selected for comparative experiments, including CS, ICS^21^, DACS^22^, VCS^23^ and DECS^10^. In the experiments, the population size $N$ of each algorithm is set to 50, the dimension $D$ of these benchmark functions is 30 and 50, and each algorithm is run on each problem for 30 times. For each run, it will be stopped if the maximum function evaluation reaches $10000\times D$. Meanwhile, the parameters of these comparative algorithms can be determined according to the original literature. For CCS algorithm, the control coefficient $\gamma$ is set to 0.2, and the discovery probability ${p}_{a}$ is set to 0.25. Additionally, the mean value (Mean) and standard deviation (Std) of the final function errors are stored for comparative analysis. For the two sets of benchmark functions with 30 dimensions, the experimental results are listed in Tables 3 and 4 respectively, and the best outputs are shown in bold. To make the conclusion more objective, Friedman test is implemented to evaluate the performance difference of these CS algorithms, and the average ranking and final ranking are provided in the numerical results. To visually compare the convergence trend, the convergent graphs of these CS algorithms on some typical benchmark functions with 30 dimensions are given in Fig. 2.

**Table 3:** Comparison of CS algorithms on the first set of benchmark functions with $D=30$.

No.	Mean/Std	CS	ICS	DACS	VCS	DECS	CCS
f1	Mean	5.07E−011	3.62E−035	4.05E−035	3.33E−026	6.26E−011	**9.40E−039**
	Std	6.17E−011	3.50E−035	4.28E−035	2.48E−026	8.24E−011	9.49E−039
f2	Mean	2.68E−005	2.22E−021	**1.17E−026**	1.70E−015	1.44E−005	6.20E−022
	Std	1.78E−005	1.25E−021	5.25E−026	1.07E−015	9.11E−006	3.96E−022
f3	Mean	4.77E−008	1.16E−006	**1.84E−012**	6.41E−008	1.90E−007	3.27E−011
	Std	2.01E−007	1.95E−006	4.42E−012	6.56E−008	7.90E−007	4.33E−011
f4	Mean	1.88E+000	5.20E−003	2.10E−003	3.20E−003	1.75E+000	**5.19E−004**
	Std	9.40E−001	2.80E−003	1.40E−003	1.70E−003	1.16E+000	2.93E−004
f5	Mean	1.80E+001	7.42E+000	2.93E+000	9.26E+000	1.86E+001	**1.98E+000**
	Std	4.72E+000	3.07E+000	1.45E+000	1.18E+000	5.43E+000	1.08E+000
f6	Mean	3.12E+003	**9.27E+002**	3.41E+003	2.85E+003	3.05E+003	2.15E+003
	Std	3.07E+002	4.52E+002	1.01E+003	7.44E+002	2.49E+002	6.12E+002
f7	Mean	4.80E+001	**3.32E+001**	5.58E+001	3.73E+001	4.46E+001	3.42E+001
	Std	9.78E+000	7.91E+000	1.31E+001	1.26E+001	7.86E+000	9.69E+000
f8	Mean	3.27E−001	1.38E−014	2.48E−014	3.03E−013	4.80E−001	**1.13E−014**
	Std	5.24E−001	3.02E−015	6.25E−015	2.09E−013	6.63E−001	2.79E−015
f9	Mean	1.70E−005	1.77E−009	**0.00E+000**	5.75E−004	5.30E−004	**0.00E+000**
	Std	4.45E−005	9.67E−009	0.00E+000	2.20E−003	1.90E−003	0.00E+000
f10	Mean	4.10E−003	2.12E−031	1.54E−020	6.23E−024	1.12E−002	**1.57E−032**
	Std	1.91E−002	6.94E−031	8.34E−020	2.23E−023	3.17E−003	5.57E−048
f11	Mean	2.60E−008	5.77E−032	1.23E−029	1.04E−024	9.67E−007	**1.35E−032**
	Std	6.67E−008	5.24E−032	2.76E−029	1.34E−024	4.83E−006	5.57E−048
Average ranking		4.9091	2.7273	2.7727	3.3636	5.2727	1.9545
Final ranking		5	2	3	4	6	1

Significant values are in bold.

**Table 4:** Comparison of CS algorithms on the second set of benchmark functions with $D=30$.

No.	Mean/Std	CS	ICS	DACS	VCS	DECS	CCS
F1	Mean	7.41E−010	**0.00E+000**	**0.00E+000**	3.03E−026	8.89E−010	**0.00E+000**
	Std	1.17E−009	0.00E+000	0.00E+000	2.58E−026	1.17E−009	0.00E+000
F2	Mean	3.14E−006	3.39E−008	**2.62E−011**	6.39E−007	3.13E−006	3.36E−010
	Std	8.84E−006	4.98E−008	8.93E−011	1.04E−006	1.06E−005	6.49E−010
F3	Mean	**1.33E+005**	1.47E+005	1.35E+005	1.48E+005	3.63E+005	1.39E+005
	Std	2.06E+005	1.53E+005	1.64E+005	1.61E+005	8.60E+005	1.97E+005
F4	Mean	2.16E+003	8.26E+000	6.88E+000	1.62E+001	2.06E+003	**1.74E+000**
	Std	2.80E+003	2.66E+001	8.66E+000	2.23E+001	1.91E+003	4.17E+000
F5	Mean	4.50E+003	**1.21E+003**	1.70E+003	1.81E+003	4.68E+003	1.38E+003
	Std	1.67E+003	5.58E+002	7.25E+002	6.81E+002	1.89E+003	3.89E+002
F6	Mean	3.57E+001	1.47E+001	8.37E+000	1.60E+001	3.18E+001	**5.43E+000**
	Std	2.87E+001	1.95E+001	1.28E+001	1.52E+001	2.22E+001	8.52E+000
F7	Mean	1.30E−003	8.90E−003	2.60E−003	**2.47E−004**	2.20E−003	5.60E−003
	Std	3.30E−003	9.50E−003	6.90E−003	1.40E−003	5.10E−003	1.01E−002
F8	Mean	2.09E+001	2.09E+001	2.09E+001	2.09E+001	2.09E+001	2.09E+001
	Std	6.78E−002	6.05E−002	6.54E−002	4.69E−002	5.47E−002	6.21E−002
F9	Mean	7.97E+001	**2.62E+001**	1.11E+002	3.12E+001	7.14E+001	4.78E+001
	Std	1.19E+001	6.36E+000	1.83E+001	7.87E+000	1.07E+001	1.08E+001
F10	Mean	1.60E+002	**5.67E+001**	1.55E+002	6.80E+001	1.24E+002	8.64E+001
	Std	3.69E+001	1.60E+001	3.76E+001	1.38E+001	2.92E+001	1.90E+001
F11	Mean	2.90E+001	2.44E+001	3.12E+001	2.43E+001	2.86E+001	**2.32E+001**
	Std	1.88E+000	3.01E+000	2.61E+000	4.08E+000	1.94E+000	2.64E+000
F12	Mean	8.15E+003	4.85E+003	1.59E+004	**3.87E+003**	1.09E+004	5.32E+003
	Std	6.38E+003	5.24E+003	1.84E+004	4.48E+003	6.75E+003	7.73E+003
F13	Mean	5.76E+000	4.05E+000	5.54E+000	**3.99E+000**	5.23E+000	4.45E+000
	Std	1.22E+000	1.19E+000	9.93E−001	1.44E+000	1.23E+000	1.69E+000
F14	Mean	1.31E+001	1.27E+001	1.29E+001	1.29E+001	1.31E+001	**1.26E+001**
	Std	1.99E−001	2.50E−001	1.95E−001	2.25E−001	1.92E−001	3.29E−001
Average ranking		4.7143	2.6429	3.6786	2.8214	4.7143	2.4286
Final ranking		6	2	4	3	6	1

Significant values are in bold.

As shown in Table 3, CCS produces better solutions in most cases. Specifically, in terms of solution quality, ICS produces promising results on f6 and f7, and it also provides high-quality results on f1, f2, f8, f10 and f11. DACS exhibits good comprehensive performance and is good at addressing f2, f3 and f9. Although VCS cannot get the highest precision solution for any problem, it produces reasonable results on the whole. Unfortunately, CS and DECS cannot perform better on any benchmark function. It should be emphasized that CCS performs best on the unimodal problems f1, f4 and f5 as well as the multimodal problems f8, f9, f10 and f11. Moreover, CCS is the second best method on f2, f3, f6 and f7. Based on the statistical results using the Friedman test, CCS ranks first with an average ranking of 1.9545, followed by ICS, DACS, VCS, CS and DECS. Therefore, CCS has better performance than other CS algorithms on the first set of benchmark functions with 30 dimensions.

From Table 4, it is observed that CCS still exhibits better convergence performance in tackling these challenging optimization problems. In more detail, CS provides the highest quality solution on F3, ICS performs best on F1, F5, F9 and F10, DACS gets promising results on F1 and F2, and VCS is superior to other algorithms on F7, F12 and F13, while DECS cannot provide the lowest mean value for any problem except F8. Similarly, CCS produces reasonable results on F1, F4, F6, F11 and F14, and it performs second best on F2 and F5. Furthermore, for the multimodal problem F8, these CS algorithms all produce the same average error. For the unimodal problem F1, ICS, DACS and CCS find the global optimal solution. In terms of the comparison results obtained by the Friedman test, CCS gets the smallest average ranking of 2.4286, followed by ICS with the average ranking of 2.6429. CS and DECS yield the largest average ranking, which means that they are losers compared with other algorithms. Therefore, it is clear that the proposed CCS is the best among these CS algorithms.

It can be seen from Fig. 2 that CCS has faster convergence speed and better search efficiency. Specifically, for the first set of functions f1, f4 and f11 as well as the second set of functions F1, F4 and F11, the convergence graph of CCS declines faster than that of other algorithms, which indicates that CCS has more advantages in terms of speed and accuracy. For f5 and F6, CCS is slightly better than other CS algorithms. Besides, for f9, CCS converges slightly slower than DACS, but they can finally find the global optimal solution. Therefore, CCS is a competitive optimization method.

Generally speaking, a promising evolutionary algorithm should also be able to produce reasonable results in tackling high-dimensional problems. To better evaluate the impact of increasing dimension on the convergence performance, the proposed CCS and five CS algorithms are compared on the basis of $D = 50$. Then, the maximum number of function evaluations is $10000 \times D$, and other parameter configurations are consistent with those in the previous experiments. For these two sets of benchmark functions, the experimental results are reported in Tables 5 and 6, respectively. The statistical results of the nonparametric Friedman test are also provided in these tables.

**Table 5:** Comparison of CS algorithms on the first set of benchmark functions with $D=50$.

No.	Mean/Std	CS	ICS	DACS	VCS	DECS	CCS
f1	Mean	2.72E−009	3.29E−032	6.89E−037	9.60E−024	7.80E−009	**5.98E−040**
	Std	2.73E−009	2.05E−032	5.16E−037	7.13E−024	7.81E−009	6.53E−040
f2	Mean	4.40E−006	8.85E−020	**1.61E−025**	7.17E−014	4.72E−006	4.03E−023
	Std	1.65E−006	3.68E−020	2.86E−025	2.65E−014	2.17E−006	2.66E−023
f3	Mean	4.54E−002	2.34E−002	**1.42E−005**	8.30E−003	4.62E−002	1.82E−005
	Std	3.75E−002	2.55E−002	2.60E−005	6.10E−003	4.15E−002	1.76E−005
f4	Mean	7.89E+000	1.00E−001	6.03E−002	1.01E−001	6.98E+000	**9.30E−003**
	Std	1.54E+000	5.31E−002	3.71E−002	5.30E−002	1.23E+000	5.20E−003
f5	Mean	5.96E+001	3.23E+001	2.24E+001	3.44E+001	5.09E+001	**1.80E+001**
	Std	2.88E+001	1.38E+001	1.00E+001	1.50E+001	2.88E+001	6.91E+000
f6	Mean	5.93E+003	**2.09E+003**	6.59E+003	4.58E+003	5.53E+003	3.58E+003
	Std	5.58E+002	5.15E+002	1.65E+003	9.70E+002	5.17E+002	6.49E+002
f7	Mean	6.44E+001	6.45E+001	7.67E+001	**4.75E+001**	5.76E+001	5.43E+001
	Std	1.67E+001	1.59E+001	1.17E+001	1.10E+001	1.31E+001	1.52E+001
f8	Mean	1.05E+000	3.05E−014	6.31E−014	2.11E−012	1.26E+000	**2.42E−014**
	Std	7.04E−001	4.10E−015	1.10E−014	9.73E−013	6.81E−001	3.58E−015
f9	Mean	2.60E−003	4.11E−004	1.19E−003	4.93E−004	3.00E−003	**0.00E+000**
	Std	4.60E−003	2.20E−003	4.00E−003	1.90E−003	4.30E−003	0.00E+000
f10	Mean	6.70E−003	6.89E−028	6.20E−003	2.10E−003	1.92E−002	**9.42E−033**
	Std	1.89E−002	2.50E−027	1.90E−002	1.14E−002	4.33E−002	1.39E−048
f11	Mean	3.91E−004	6.66E−029	1.70E−030	1.44E−022	1.10E−003	**1.35E−032**
	Std	2.00E−003	1.40E−028	6.97E−030	1.41E−022	4.30E−003	5.57E−048
Average ranking		5.0909	2.6364	3.1364	2.9545	5.3636	1.8182
Final ranking		5	2	4	3	6	1

Significant values are in bold.

**Table 6:** Comparison of CS algorithms on the second set of benchmark functions with $D=50$.

No.	Mean/Std	CS	ICS	DACS	VCS	DECS	CCS
F1	Mean	7.46E−008	**0.00E+000**	**0.00E+000**	9.90E−024	1.36E−007	**0.00E+000**
	Std	1.18E−007	0.00E+000	0.00E+000	7.26E−024	1.47E−007	0.00E+000
F2	Mean	2.14E+000	7.30E−003	**1.22E−004**	1.10E−001	2.48E+000	3.55E−004
	Std	1.91E+000	6.60E−003	9.76E−005	7.25E−002	1.70E+000	3.14E−004
F3	Mean	3.17E+007	9.63E+006	**7.88E+006**	1.51E+007	3.39E+007	8.82E+006
	Std	1.95E+007	4.71E+006	6.78E+006	8.92E+006	1.82E+007	4.97E+006
F4	Mean	2.86E+004	3.25E+003	6.12E+003	8.65E+003	2.77E+004	**2.10E+003**
	Std	8.60E+003	1.95E+003	3.22E+003	3.54E+003	9.29E+003	1.26E+003
F5	Mean	9.67E+003	**4.53E+003**	6.16E+003	5.88E+003	1.06E+004	5.08E+003
	Std	1.89E+003	6.58E+002	1.47E+003	1.30E+003	2.33E+003	1.39E+003
F6	Mean	1.10E+002	4.50E+001	2.93E+001	4.23E+001	8.38E+001	**2.27E+001**
	Std	5.61E+001	2.55E+001	2.62E+001	2.47E+001	4.14E+001	2.36E+001
F7	Mean	1.13E−002	5.00E−003	**2.20E−003**	4.50E−003	9.80E−003	4.10E−003
	Std	1.21E−002	9.70E−003	5.30E−003	8.70E−003	9.20E−003	6.90E−003
F8	Mean	2.11E+001	2.11E+001	2.11E+001	2.11E+001	2.11E+001	2.11E+001
	Std	3.74E−002	4.17E−002	4.94E−002	8.04E−002	4.65E−002	3.36E−002
F9	Mean	1.64E+002	**5.51E+001**	2.40E+002	6.61E+001	1.58E+002	9.58E+001
	Std	1.70E+001	1.44E+001	3.30E+001	2.06E+001	2.36E+001	2.02E+001
F10	Mean	4.67E+002	**1.23E+002**	4.32E+002	1.39E+002	4.57E+002	2.19E+002
	Std	5.63E+001	3.09E+001	8.90E+001	2.03E+001	9.56E+001	4.63E+001
F10	Mean	5.38E+001	4.44E+001	5.87E+001	4.47E+001	5.33E+001	**4.30E+001**
	Std	2.61E+000	8.44E+000	2.85E+000	6.62E+000	2.79E+000	6.92E+000
F12	Mean	5.77E+004	4.07E+004	2.61E+005	4.88E+004	5.06E+004	**3.21E+004**
	Std	4.24E+004	2.99E+004	2.83E+005	4.58E+004	4.10E+004	2.46E+004
F13	Mean	1.27E+001	7.41E+000	1.16E+001	**6.29E+000**	1.26E+001	7.30E+000
	Std	2.32E+000	2.51E+000	1.60E+000	1.72E+000	2.55E+000	3.32E+000
F14	Mean	2.26E+001	2.24E+001	2.24E+001	2.24E+001	2.26E+001	**2.23E+001**
	Std	3.12E−001	2.98E−001	2.52E−001	3.31E−001	2.48E−001	2.66E−001
Average ranking		5.2857	2.5000	3.3571	2.9286	5.0000	1.9286
Final ranking		6	2	4	3	5	1

Significant values are in bold.

It can be observed from Table 5 that CCS still maintains stronger competitiveness in addressing these 50 dimensional benchmark functions. According to the quality of the solution, CCS produces the highest accuracy solution on 7 problems, namely f1, f4, f5, f8, f9, f10 and f11. ICS performs best on f6, DACS is good at solving the unimodal problems f2 and f3, VCS is better than other algorithms on f7. However, DACS and VCS have the defect of unstable search when dealing with the multimodal problems f9 and f10. Also, neither CS nor DECS can get the best results for any of these 50 dimensional test functions. In terms of the comparison results of the Friedman test, CCS produces the best comprehensive performance, followed by ICS. DECS is defeated by other algorithms because its robustness in solving different optimization problems needs to be further strengthened. Therefore, the presented CCS has better performance than other CS algorithms in tackling these high-dimensional basic problems.

As indicated in Table 6, CCS achieves better convergence performance for these 50 dimensional challenging problems. For example, ICS produces reasonable results on F1, F5, F9 and F10, DACS gets the lowest mean value on F1, F2, F3 and F7, but it is not competitive in solving F9, F10, F11, F12 and F13. VCS provides the highest quality result on F13, but CS and DECS are still not good at dealing with these complex optimization problems. Similarly, CCS produces promising results on F1, F4, F6, F11, F12 and F14, and it performs second best on F2, F3, F5, F7 and F13. Moreover, it is difficult to determine which algorithm is more suitable for solving F8. Based on the ranking results obtained by the Friedman test, these CS algorithms are sorted as follows: CCS, ICS, VCS, DACS, DECS and CS. Apparently, CCS beats its peers on these challenging benchmark functions. From the above observations, it can be found that CCS is more competitive than other CS algorithms.

**Figure 3:**
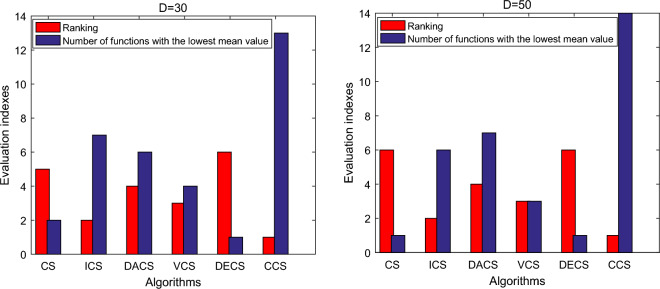
Statistical results of tackling these 25 benchmark functions using different algorithms.

Additionally, for these 25 benchmark functions with 30 and 50 dimensions, the statistical results in terms of the number of functions with the lowest mean value produced by different algorithms and their final ranking are plotted in Fig. 3. Obviously, compared to other algorithms, CCS exhibits the most competitive performance in addressing these optimization problems with different dimensions.

### Comparison with non-CS algorithms

In this section, CCS is compared with five non-CS algorithms on the two sets of benchmark functions with 30 dimensions. These algorithms include AEFA^24^, FPA^25^, SCA^26^, BOA^27^ and WOA^28^. The population size of each algorithm is 50, and the maximum number of function evaluations is set to 300,000. Moreover, each algorithm is run 30 times, and the average value and standard deviation of each method on each test function are given in Tables 7 and 8. The best average value is marked in bold. To further comprehensively compare the performance of CCS and these non-CS algorithms, the Friedman test is conducted, and the statistical results are provided in the last two rows of these tables.

**Table 7:** Comparison of AEFA, FPA, SCA, BOA, WOA and CCS on the first set of benchmark functions with $D=30$.

No.	Mean/Std	AEFA	FPA	SCA	BOA	WOA	CCS
f1	Mean	5.96E−025	8.09E−009	1.58E−037	1.01E−022	**0.00E+000**	9.40E−039
	Std	1.00E−025	6.09E−009	5.37E−037	5.53E−022	0.00E+000	9.49E−039
f2	Mean	3.48E−012	4.36E−004	1.88E−037	3.53E−026	**0.00E+000**	6.20E−022
	Std	4.05E−013	2.16E−004	9.96E−037	4.07E−026	0.00E+000	3.96E−022
f3	Mean	9.29E+001	5.24E−006	7.12E+001	**7.92E−031**	2.60E−001	3.27E−011
	Std	5.48E+001	5.05E−006	2.89E+002	5.03E−031	8.44E−001	4.33E−011
f4	Mean	4.10E−013	2.20E+000	1.78E−001	**1.32E−028**	5.48E+001	5.19E−004
	Std	4.05E−014	1.14E+000	8.54E−001	6.06E−029	1.40E+001	2.93E−004
f5	Mean	2.38E+001	2.00E+001	2.75E+001	2.90E+001	1.43E+001	**1.98E+000**
	Std	1.50E−001	4.66E+000	6.59E−001	1.11E−002	1.07E+000	1.08E+000
f6	Mean	9.92E+003	3.06E+003	8.21E+003	5.31E+003	2.50E+003	**2.15E+003**
	Std	3.72E+002	1.96E+002	2.80E+002	1.12E+003	1.61E+003	6.12E+002
f7	Mean	1.52E+001	5.46E+001	4.09E−007	**0.00E+000**	1.99E+001	3.42E+001
	Std	4.94E+000	9.89E+000	2.21E−006	**0.00E+000**	4.34E+001	9.69E+000
f8	Mean	6.08E−013	1.42E+000	9.19E+000	**8.88E−016**	5.03E−015	1.13E−014
	Std	7.36E−014	8.06E−001	9.63E+000	0.00E+000	1.89E−015	2.79E−015
f9	Mean	3.29E−004	2.34E−005	4.76E−007	**0.00E+000**	**0.00E+000**	**0.00E+000**
	Std	1.80E−003	3.41E−005	2.58E−006	0.00E+000	0.00E+000	0.00E+000
f10	Mean	3.50E−003	1.84E−002	3.33E−001	1.46E+000	1.04E−002	**1.57E−032**
	Std	1.89E−002	5.74E−002	3.88E−002	2.07E−001	3.16E−002	5.57E−048
f11	Mean	5.34E−004	7.57E−006	1.97E+000	3.00E+000	2.64E−001	**1.35E−032**
	Std	2.20E−003	1.70E−005	1.15E−001	1.10E−003	1.17E+000	5.57E−048
Average ranking		3.8182	4.3636	4.2273	3.2273	3.1818	2.1818
Final ranking		4	6	5	3	2	1

Significant values are in bold.

**Table 8:** Comparison of AEFA, FPA, SCA, BOA, WOA and CCS on the second set of benchmark functions with $D=30$.

No.	Mean/Std	AEFA	FPA	SCA	BOA	WOA	CCS
F1	Mean	6.12E−025	4.67E−008	7.00E+001	5.02E+004	7.63E−006	**0.00E+000**
	Std	9.91E−026	5.45E−008	2.68E+001	8.89E+003	2.13E−005	0.00E+000
F2	Mean	1.62E+003	1.61E−004	2.33E+003	5.56E+004	1.90E+004	**3.36E−010**
	Std	4.62E+002	2.76E−006	6.55E+002	1.16E+004	4.41E+003	6.49E−010
F3	Mean	2.14E+006	**6.73E+001**	8.43E+006	1.08E+009	3.65E+006	1.39E+005
	Std	8.57E+005	2.10E+002	1.66E+006	5.10E+008	1.88E+006	1.97E+005
F4	Mean	1.47E+004	3.76E+003	5.26E+003	1.33E+005	1.13E+005	**1.74E+000**
	Std	3.05E+003	2.40E+003	1.83E+003	6.02E+004	3.01E+004	4.17E+000
F5	Mean	1.39E+003	**1.28E+003**	2.68E+003	3.03E+004	2.71E+004	1.38E+003
	Std	2.46E+002	6.10E+002	3.66E+002	6.01E+003	5.02E+003	3.89E+002
F6	Mean	1.35E+003	4.78E+001	1.05E+005	7.83E+009	1.05E+002	**5.43E+000**
	Std	2.10E+003	4.52E+001	8.75E+004	5.09E+009	1.56E+002	8.52E+000
F7	Mean	5.06E+002	**1.60E−003**	1.21E+001	2.88E+003	6.25E−002	5.60E−003
	Std	6.79E+001	3.40E−003	8.75E+000	7.86E+002	3.84E−002	1.01E−002
F8	Mean	**2.01E+001**	2.09E+001	2.09E+001	2.09E+001	2.03E+001	2.09E+001
	Std	6.35E−002	6.19E−002	5.34E−002	4.01E−002	6.78E−002	6.21E−002
F9	Mean	**1.77E+001**	6.96E+001	6.65E+001	3.12E+002	2.09E+002	4.78E+001
	Std	4.51E+000	7.49E+000	8.57E+000	2.57E+001	3.97E+001	1.08E+001
F10	Mean	**1.68E+001**	1.94E+002	9.16E+001	4.47E+002	4.06E+002	8.64E+001
	Std	4.48E+000	4.22E+001	1.05E+001	5.15E+001	6.68E+001	1.90E+001
F11	Mean	**1.37E−004**	2.86E+001	2.41E+001	4.07E+001	4.07E+001	2.32E+001
	Std	6.63E−004	1.27E+000	1.94E+000	1.77E+000	2.41E+000	2.64E+000
F12	Mean	**3.00E+000**	1.69E+004	2.51E+004	1.85E+006	6.79E+004	5.32E+003
	Std	5.06E+000	6.02E+003	1.04E+004	2.70E+005	4.20E+004	7.73E+003
F13	Mean	5.25E+000	5.63E+000	8.55E+000	2.46E+002	2.45E+001	**4.45E+000**
	Std	1.27E+000	1.10E+000	1.14E+000	1.09E+002	1.01E+001	1.69E+000
F14	Mean	1.37E+001	1.30E+001	**1.24E+001**	1.36E+001	1.35E+001	1.26E+001
	Std	1.82E−001	1.91E−001	3.58E−001	2.09E−001	3.29E−001	3.29E−001
Average ranking		2.6429	2.6071	3.7500	5.7857	4.3214	1.8929
Final ranking		3	2	4	6	5	1

Significant values are in bold.

From Table 7, it can be observed that CCS exhibits better comprehensive performance compared with these advanced evolutionary algorithms. In more detail, BOA is significantly better than others on f3, f4, f7 and f8, and it also provides the global optimal value on f9. WOA yields the best search results on f1, f2 and f9, but it loses its advantages in addressing f4, f10 and f11. It should be noted that AEFA, FPA and SCA cannot produce the best results for any problem. Further, CCS provides promising comprehensive results, especially in tackling f5, f6, f9, f10 and f11. In accordance with the average ranking of these algorithms, CCS gets the smallest average ranking of 2.1818, followed by WOA, BOA, AEFA, SCA and FPA in rising direction, which means that CCS is the best overall.

As shown in Table 8, these algorithms exhibit different advantages in solving different benchmark functions. In terms of solution accuracy, AEFA gets the solutions with higher accuracy on F8, F9, F10, F11 and F12, FPA performs best on F3, F5 and F7, SCA produces better performance on F14. However, BOA and WOA are not good at solving these challenging optimization problems, and they cannot produce the highest quality results for any problem. Also, CCS outperforms others on F1, F2, F4, F6 and F13, and it is the second best approach on F3, F5, F7, F9, F10, F11, F12 and F14. According to the average ranking and final ranking, CCS is significantly superior to these non-CS algorithms. Considering all the above analysis, it can be easily found that the proposed CCS algorithm has better comprehensive performance.

### Chaotic time series prediction

In a complex system, the digital sequence obtained from the observation variables based on the time order is called the time series, which can reflect the dynamic properties of the system. The time series obtained from chaotic systems is called a chaotic time series, which is a dataset with nonlinear characteristics and contains rich system dynamic information. In practical application, the prediction of chaotic time series has always been a research hotspot^29^.

To further investigate the competitiveness of CCS algorithm, two typical chaotic systems, Lorenz and Mackey–Glass^30^, are used for numerical simulation and comparison. In this work, the three-layer feed-forward neural network is used as the prediction model of chaotic time series, CCS and CS are used as learning algorithms to independently determine the initial weights and thresholds of the neural network.

The first chaotic system is the Lorenz system described as follows:

11 $$ \begin{gathered} \dot{x}_{1} \left( t \right) = m\left( {x_{2} \left( t \right) - x_{1} \left( t \right)} \right) \hfill \\ \dot{x}_{2} \left( t \right) = nx_{1} \left( t \right) - x_{2} \left( t \right) - x_{1} \left( t \right)x_{3} \left( t \right) \hfill \\ \dot{x}_{3} \left( t \right) = x_{1} \left( t \right)x_{2} \left( t \right) - px_{3} \left( t \right) \hfill \\ \end{gathered} $$

where $$m = 10$$, $$n = 28$$ and $$p = 8/3$$.

The second chaotic system is the Mackey–Glass system, which is formulated as follows:

12 $$ \dot{x}\left( t \right) = - mx\left( t \right) + \frac{{nx\left( {t - 17} \right)}}{{1 + x^{10} \left( {t - 17} \right)}} $$

where $$m = 0.1$$ and $$n = 0.2$$.

**Figure 4:**
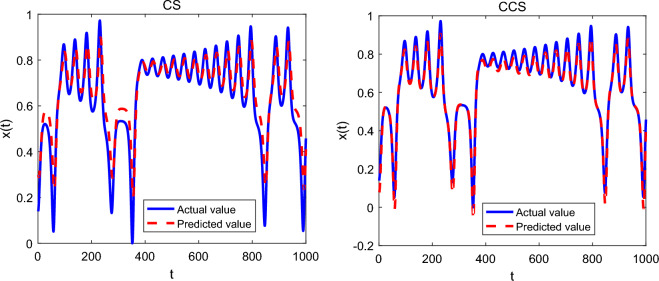
Comparison of CCS and CS on the Lorenz time series.

**Figure 5:**
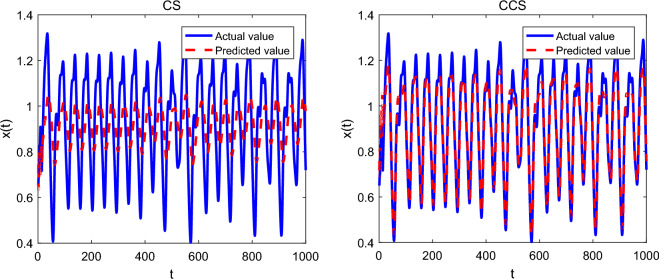
Comparison of CCS and CS on the Mackey–Glass time series.

In the experiments, the population size of CCS and CS algorithms is 30, and the number of repeated runs on each chaotic time series is 25. For the Lorenz chaotic system, the maximum number of iterations is set to 300, and the number of neurons from the input layer to the output layer of the feed-forward neural network is 3, 6 and 1 respectively. For the Mackey–Glass chaotic system, the maximum number of iterations is set to 500, and the number of nodes in the input layer, hidden layer and output layer is 4, 6 and 1 respectively. The experimental results in terms of mean value and standard deviation of the squared errors are shown in Table 9, and the performance comparison on different chaotic systems is given in Figs. 4 and 5.

**Table 9:** Experimental results of CS and CCS algorithms.

System	CS		CCS	
	Mean value	Standard deviation	Mean value	Standard deviation
Lorenz	1.16E−002	3.09E−003	4.18E−003	1.75E−003
Mackey–Glass	4.72E−002	1.84E−003	1.50E−002	8.39E−003

Observed from Table 9, for the Lorenz and Mackey–Glass chaotic systems, CCS provides the smallest mean error of 4.18E–003 and 1.50E–002, respectively. In other words, CCS has higher prediction accuracy than CS algorithm. As reported in Figs. 4 and 5, the predicted value of CCS for each chaotic time series is closer to the actual value of the corresponding system. In conclusion, the above observations show that the proposed CCS algorithm has good search ability and can improve the prediction accuracy of chaotic time series.

## Conclusions

To enhance the convergence performance of CS algorithm in addressing various optimization problems, a new CS algorithm based on cloud model is proposed to adaptively determine the step size factor. First, the set of step size factors is considered as a cloud, and three numerical characteristics in the cloud model are defined by using the individual fitness information in the population. Then, the membership degree of each step size factor is calculated. Finally, an exponential function is designed to adaptively configure the step size factor, so as to enhance the versatility and robustness of the algorithm. To evaluate the effectiveness of CCS algorithm, a large number of experiments are conducted on two sets of benchmark functions to compare it with other CS algorithms and five advanced evolutionary algorithms. Further, the non-parametric statistical Friedman test is carried out for comprehensive comparison and analysis. Experimental results indicate that CCS algorithm has strong competitiveness in terms of search accuracy and convergence rate. Besides, the proposed CCS is also a promising method in predicting two chaotic time series. However, CCS algorithm also has some limitations, such as not being able to produce the most promising results for all benchmark functions. In the future, an adaptive configuration scheme for the control coefficient will be explored to enhance the robustness of solving different optimization problems, and more real-world applications will be considered to further investigate the effectiveness of CCS algorithm.

## Data Availability

The data obtained through the experiments are available from the corresponding author on reasonable request.
